# Standardization of a Continuous Assay for Glycosidases and Its Use for Screening Insect Gut Samples at Individual and Populational Levels

**DOI:** 10.3389/fphys.2017.00308

**Published:** 2017-05-12

**Authors:** Gerson S. Profeta, Jessica A. S. Pereira, Samara G. Costa, Patricia Azambuja, Eloi S. Garcia, Caroline da Silva Moraes, Fernando A. Genta

**Affiliations:** ^1^Instituto Oswaldo Cruz, Fundação Oswaldo Cruz (FIOCRUZ)Rio de Janeiro, Brazil; ^2^Instituto Nacional de Ciência e Tecnologia em Entomologia MolecularRio de Janeiro, Brazil

**Keywords:** glycoside hydrolase, glycosidase, fluorescent assay, insect digestion, *Rhodnius prolixus*, *Lutzomyia longipalpis*

## Abstract

Glycoside Hydrolases (GHs) are enzymes able to recognize and cleave glycosidic bonds. Insect GHs play decisive roles in digestion, in plant-herbivore, and host-pathogen interactions. GH activity is normally measured by the detection of a release from the substrate of products as sugars units, colored, or fluorescent groups. In most cases, the conditions for product release and detection differ, resulting in discontinuous assays. The current protocols result in using large amounts of reaction mixtures for the obtainment of time points in each experimental replica. These procedures restrain the analysis of biological materials with limited amounts of protein and, in the case of studies regarding small insects, implies in the pooling of samples from several individuals. In this respect, most studies do not assess the variability of GH activities across the population of individuals from the same species. The aim of this work is to approach this technical problem and have a deeper understanding of the variation of GH activities in insect populations, using as models the disease vectors *Rhodnius prolixus* (Hemiptera: Triatominae) and *Lutzomyia longipalpis* (Diptera: Phlebotominae). Here we standardized continuous assays using 4-methylumbelliferyl derived substrates for the detection of α-Glucosidase, β-Glucosidase, α-Mannosidase, N-acetyl-hexosaminidase, β-Galactosidase, and α-Fucosidase in the midgut of *R. prolixus* and *L. longipalpis* with results similar to the traditional discontinuous protocol. The continuous assays allowed us to measure GH activities using minimal sample amounts with a higher number of measurements, resulting in data that are more reliable and less time and reagent consumption. The continuous assay also allows the high-throughput screening of GH activities in small insect samples, which would be not applicable to the previous discontinuous protocol. We applied continuous GH measurements to 90 individual samples of *R. prolixus* anterior midgut homogenates using a high-throughput protocol. α-Glucosidase and α-Mannosidase activities showed the normal distribution in the population. β-Glucosidase, β-Galactosidase, N-acetyl-hexosaminidase, and α-Fucosidase activities showed non-normal distributions. These results indicate that GHs fluorescent-based high-throughput assays apply to insect samples and that the frequency distribution of digestive activities should be considered in data analysis, especially if a small number of samples is used.

## Introduction

Glycosidases are key enzymes in central and intermediate metabolic pathways. They release terminal sugars from glycosides as oligo- or disaccharides, aryl, and alkyl glycosides. They may also have activity against a wide array of glycoconjugates, as polysaccharides, glycoproteins, or glycolipids (Terra and Ferreira, 2005).

The enzymatic detection of glycosidases is generally based on the secondary detection of released monosaccharides (as glucose). Alternatively, colorimetric or fluorogenic groups from synthetic substrates as *p*-nitrophenol or methyl umbelliferyl derived glycosides are measured after release (Gontijo et al., 1998; Jacobson et al., 2007; Moraes et al., 2012; Vale et al., 2012; Moreira et al., 2015). In most cases, this results in obligatory discontinuous protocols. The indirect detection of sugars may need further incubation with reagents as Glucose Oxidase (Baker, 1991) or conditions for the detection of absorbance/fluorescence of released groups may significantly differ from the conditions used for the reaction of glycosidase with its substrate (Baker and Woo, 1992).

Insect digestive glycosidases are essential enzymes for nutrition, regardless of taxonomic order or diet. These enzymes are essential for final digestion of sugars and have important roles in the interaction of insects with plants and pathogens (Terra and Ferreira, 2005). GH activities have been extensively studied in different insect orders, including agricultural pests and disease vectors. They are responsible for the breaking down of molecules of plant and animal sources (Dillon and el-Kordy, 1997; Gontijo et al., 1998; Jacobson and Schlein, 2001; Terra and Ferreira, 2005; Jacobson et al., 2007; Mury et al., 2009; Fonseca et al., 2010; Ghadamyari et al., 2010; Moraes et al., 2012; Vale et al., 2012; Sellami and Jamoussi, 2016).

Glycosidase activities vary according to the insect diet. Dillon and el-Kordy (1997) have shown differences in profiles of midgut α-glucosidase in sugar and blood fed *Phlebotomus langeroni* sand flies. According to these authors, the results may be related to the variation in the digestion of these two types of meals. Sugar is kept into the crop and released continuously to the midgut, and blood passes directly to this compartment, the place where the digestions of these diets are accomplished. Digestive glycosidases were also found in *L. longipalpis* larvae, and in this case, they are probably involved in degradation of bacterial and fungal cell walls (Moraes et al., 2012; Vale et al., 2012).

Furthermore, *R. prolixus* α-glucosidases have an important role in the detoxification of heme after a blood meal. Mury et al. (2009) have verified an increase in α-glucosidase activity and release of heme in the midgut of *R. prolixus* fed with hemin-enriched diet. Previous works (Ribeiro and Pereira, 1984; Ferreira et al., 1988a,b; Terra et al., 1988) have also shown the presence of several GHs on different nymphal stages induced by blood meal through discontinuous assays.

Discontinuous assays are routinely used for insect gut glycosidases mainly because their acidic optimum pH range is not compatible with the direct measurement of released *p*-nitrophenyl or methyl umbelliferyl groups, which give best signals in alkaline conditions. Due to the small amounts of protein that are obtained from most insect samples, coupled with the high amounts of enzyme needed for discontinuous enzyme assays, high-throughput screening is not available for the study, and characterization of many insect glycosidases. One consequence of these limitations is that GH activities in insects are commonly obtained from pools of samples from several individuals, and described as means ± SEM of few experimental measurements. Because of that, most studies do not assess the variability of GH activities across the population, and the described values do not account for subsets or extreme variations which can occur naturally in a biological species or as a consequence of different physiological or pathological individual conditions.

The aim of this work is to approach this technical problem and have a deeper understanding of the variation of GH activities in insect populations, using as models the disease vectors *R. prolixus* (Hemiptera: Triatominae) and *Lutzomyia longipalpis* (Diptera: Phlebotominae). We tested the use of continuous assays for the detection and measurement of acidic digestive glycosidases in these two insect species. Standardization of assay conditions allowed us to obtain continuous activity measurements similar to the discontinuous protocol. We applied those techniques for the measurement of six glycosidases (α-Glucosidase, β-Glucosidase, α-Mannosidase, N-acetyl-hexosaminidase, β-Galactosidase, and α-Fucosidase) in *R. prolixus* and α-Glucosidase in *L. longipalpis*. Additionally, we conducted high-throughput measurements of these enzymes in *R. prolixus*, which allowed us to study simultaneously the population distribution of all these activities using individual insect samples.

## Materials and methods

### Chemicals

The substrates used in this study were all from Sigma-Aldrich, namely 4-methylumbelliferyl α-D-glucopyranoside (cat. No. M9766, for α-glucosidase); 4-methylumbelliferyl α-D-mannopyranoside (cat. No. M3657, for α-mannosidase); 4-methylumbelliferyl β-D-glucopyranoside (cat. No. M3633, for β-glucosidase); 4-methylumbelliferyl β-D-galactopyranoside (cat. No. M1633, for β-galactosidase); 4-methylumbelliferyl N-acetyl-β-glucosaminide (cat. No. M2133, for N-acetyl- β-hexosaminidase), and 4-methylumbelliferyl α-L-fucopyranoside (cat. No M8527, for α-fucosidase). Stock solutions were prepared in dimethyl sulfoxide (DMSO) at 10 mM, α-glucosidase was from *Saccharomyces cerevisiae* (cat. No. G5003). All reagents used in this work are analytical grade (Sigma-Aldrich).

### Insects

We used adult males of *R. prolixus* (Hemiptera: Reduviidae), obtained from our insectary at FIOCRUZ (Rio de Janeiro), maintained at 28°C and 75% relative humidity. Insects were regularly fed with defibrinated rabbit blood (Azambuja and Garcia, 1997). For standardization of continuous assays, insects were dissected 5 days after blood feeding. For populational high-throughput measurements, adults were synchronized by collecting 5th instar nymphs that molted to adults in a 1–2 days time range. These adults were starved and kept without any source of food or water for 40 days until dissection and activity measurements.

*L. longipalpis* originally from Jacobina (Bahia state, Brazil) were kept as described in Moraes et al. (2014). We used adult males fed with sucrose 70% (w/v) for all the experiments described. We used recently emerged adults with ages ranging from 4 to 7 days, with sucrose meals offered *ad libitum* during 4–5 days.

### Effect of pH on fluorescence of 4-methylumbelliferone

Fluorescence of 4-methylumbelliferone was measured in different 0.2 M buffers ranging from pH 2 to 11, namely Glycine (pH 2–3), Sodium Acetate (pH 4), MES (pH 5–7), Cacodylate (pH 8), Tris (pH 9), and Sodium Carbonate (pH 10–11), in the amount range from 62.4 to 624 picomoles.

### Preparation of samples

Insects were immobilized by placing them on the ice and dissected in cold 0.9% (w/v) NaCl. For *R. prolixus*, we separated the Anterior Midgut (AM) and homogenized the whole organ in 0.6 mL 0.9% NaCl. After that, contents and tissues were separated by centrifugation at 4°C, 5,000 X g, for 5 min (Houseman and Downe, 1981). For *L. longipalpis*, we separated the whole gut from the rest of body (head plus carcass) and homogenized them in 50 μL of citrate phosphate buffer 50 mM pH 6 containing Triton X-100 1% (v/v) for better solubilization.

### Enzyme assays

We mixed 25 μL of the sample at appropriate dilution with 74 μL of 0.2 M sodium acetate buffer pH 4.5 and 1 μL of MU-glycoside stock solution (item 2.1). Substrate hydrolysis was monitored at 30°C in a spectrofluorometer Spectramax Gemini XPS (Molecular Devices) on λ_Ex_ = 355 nm and λ_Em_ = 460 nm. Glycosidase activities were expressed in micro units (μU) per insect. One unit (U) correspond to the enzyme quantity that process 1 μmol of the substrate for a minute (IUBMB, 1992). In continuous assays, we measured fluorescence intensity for 60 min with reading each minute. In discontinuous assays, reaction mixtures were incubated at 30°C for 40 min, and 200 μL 0.75 M sodium carbonate buffer pH 10 were added to stop reaction every 10 min, and end point measurements were performed for all time points.

### Statistical analysis

Graphs and data analysis were performed with the software GraphPad Prism version 5.01 for Windows. Lilliefors-van Soest and Shapiro–Wilks normality tests were employed for verification of Gaussian distributions. Data for activities that showed a normal distribution in the population were submitted to parametric unpaired Student's *t*-test, and data for enzymes that did not present Gaussian distribution were submitted to non-parametric Mann–Whitney tests.

## Results

**Figure 1:**
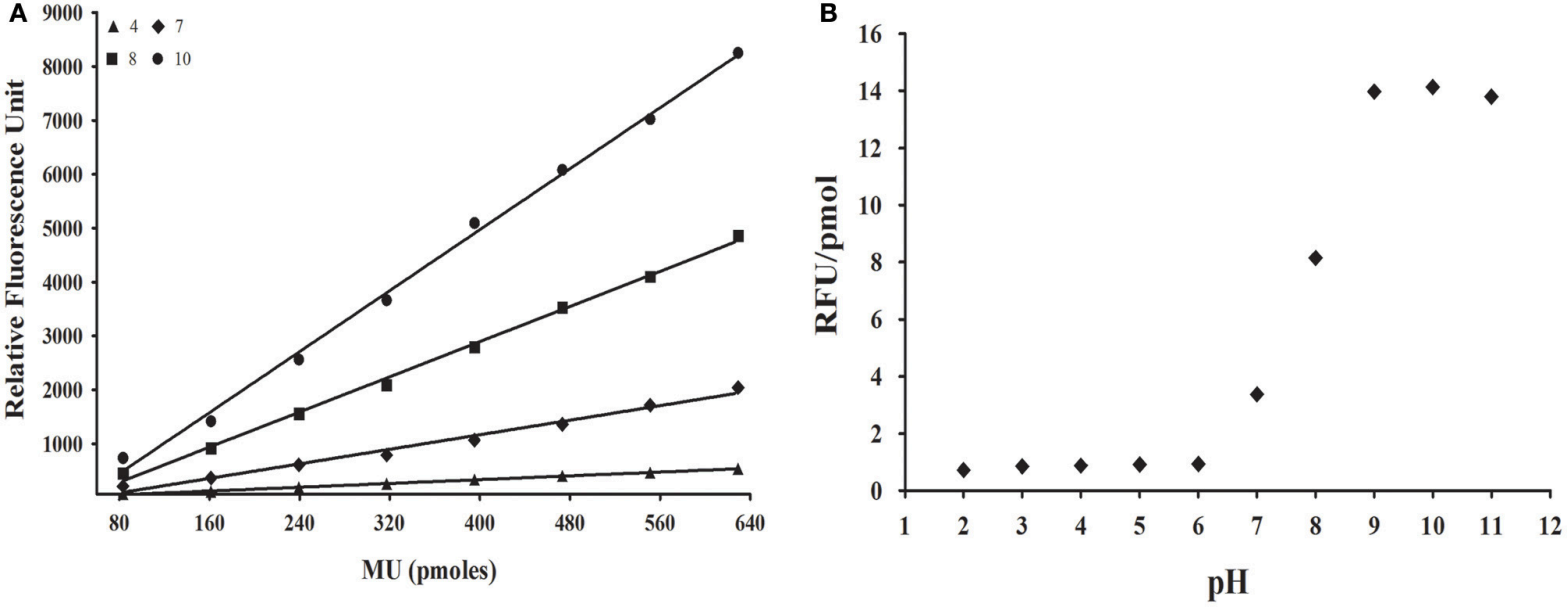
**Effect of pH on the fluorescence of methylumbelliferone. (A)** Illustrative standard curves of methylumbelliferone vs. fluorescence in four different pH values. **(B)** Dependence of fluorescence response [slope of standard curves as depicted in **(A)**, in RFU/pmol] of methylumbelliferone in the pH range 2–11. MU, Methylumbelliferone.

Initially, we investigated if fluorescence of the 4-methylumbelliferone (MU) group might be detected in acidic pHs, because glycosidase assays for *R. prolixus* and *L. longipalpis* are routinely done at pHs 4.5 and 6, respectively. For that, we performed standard curves for MU in pHs ranging from 2 to 11 (Figure 1A). We observed a detectable and significant response in all tested pHs, with smaller fluorescence values in acidic pHs (below 6) and maximum fluorescence in strongly alkaline conditions (above 9). The change in fluorescence response along pH 6–9 suggests that the MU group has a pKa around 8 (Figure 1B). Nevertheless, even in acidic conditions, the fluorescence response of MU was linear with concentration, which allowed us to measure the release of this group at the optimum conditions for the desired target glycosidases above.

**Figure 2:**
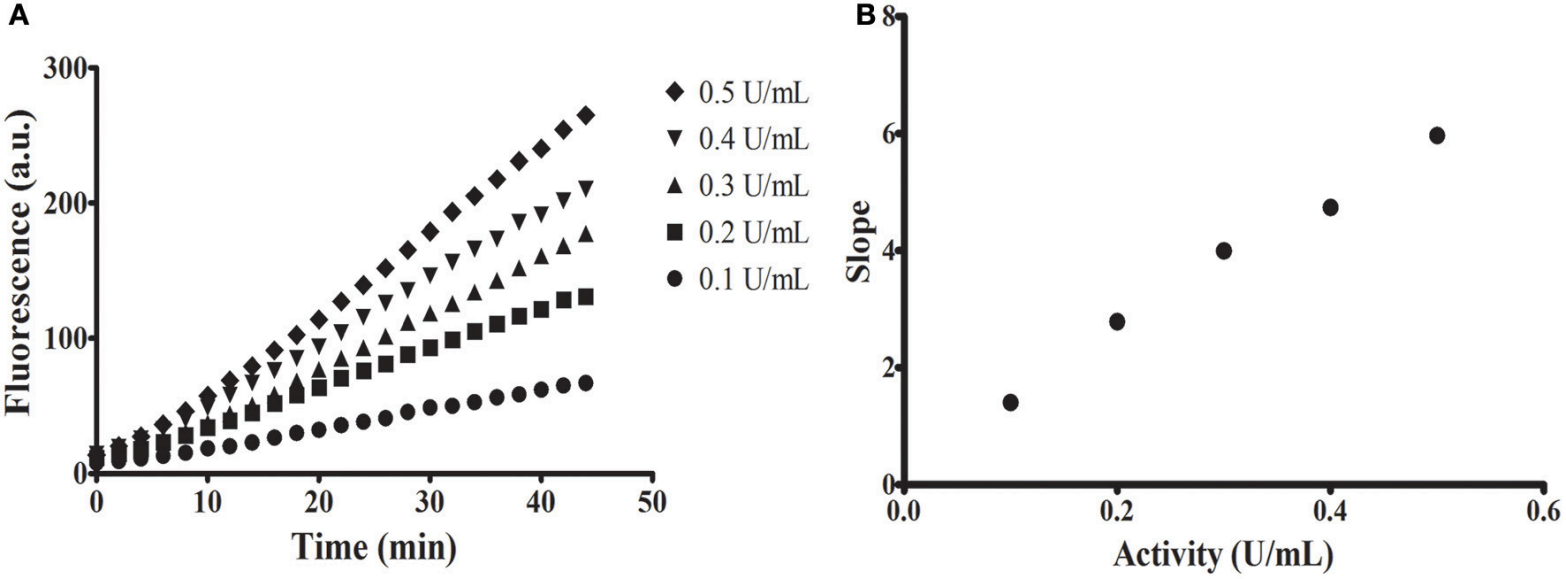
**Continuous assays using commercial α-glucosidase from Sigma. (A)** Increase in fluorescence with time using different concentrations of enzyme. **(B)** Linear relationship between measured activity [slope of assays in **(A)**] and concentration of enzyme (in units as defined by the fabricant).

To confirm if the release of the MU group by a glycosidase might be followed continuously in acidic conditions, we performed test assays with a commercial α-glucosidase (Sigma) in pH 6 (Figure 2). All assays performed showed proportionality of fluorescence with time with significant linear correlations (Figure 2A). Additionally, we observed proportionality of the measured activity with increasing concentrations of enzyme (Figure 2B).

**Figure 3:**
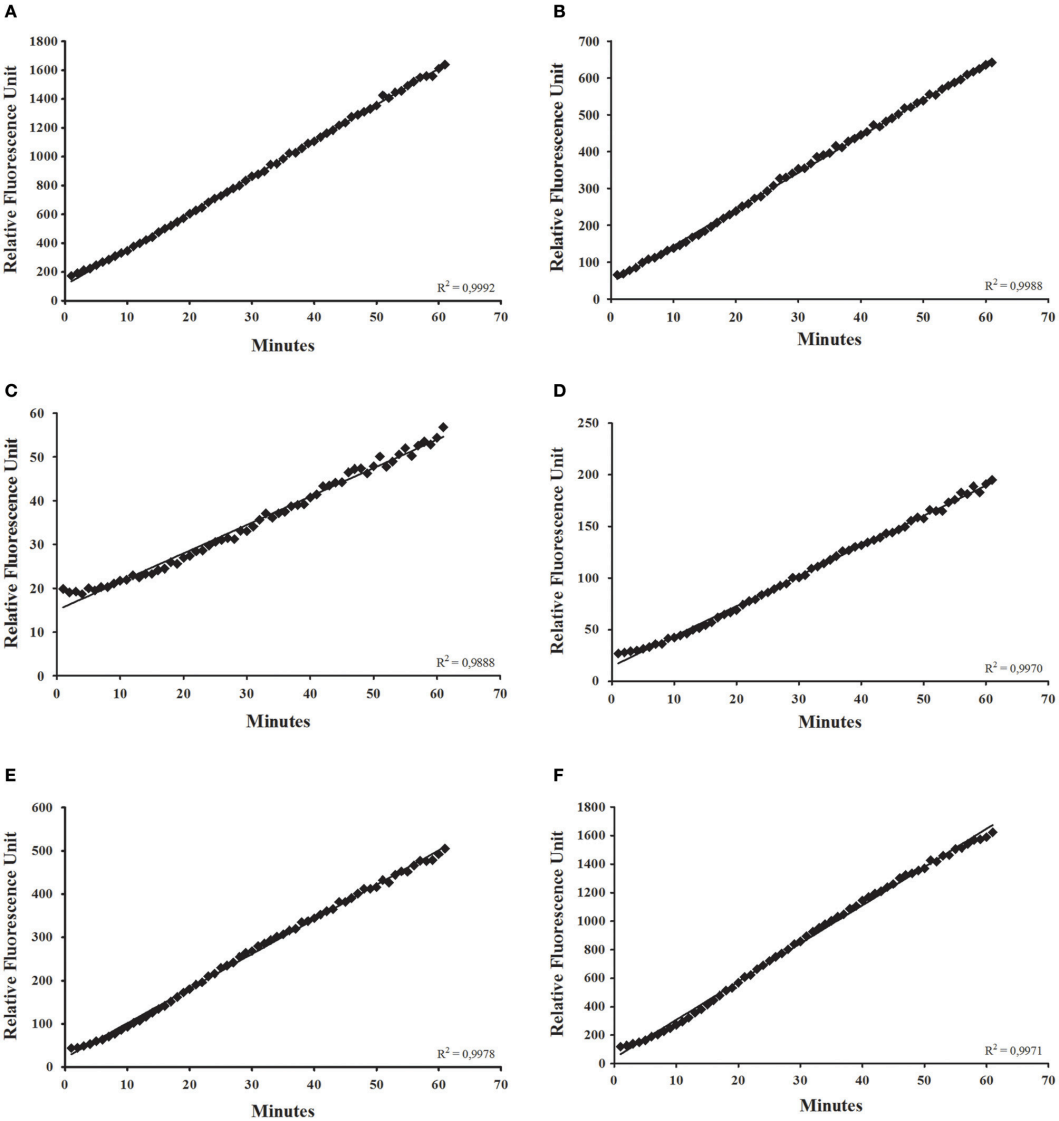
**Continuous glycosidase assays using the anterior midgut epithelium of ***Rhodnius prolixus*** as an enzyme source, showing the initial velocity measurements for (A)** α-glucosidase; **(B)** α-mannosidase; **(C)** β-glucosidase; **(D)** β-galactosidase; **(E)** β-N-acetyl-glucosaminidase; **(F)** α-fucosidase.

We applied the continuous protocol for the detection of diverse glycosidases in *R. prolixus* anterior midgut tissue homogenates (Figure 3). We obtained linear assays for α-glucosidase (Figure 3A), α-mannosidase (Figure 3B), β-glucosidase (Figure 3C), β-galactosidase (Figure 3D), β-N-acetyl-hexosaminidase (Figure 3E), and α-fucosidase activities (Figure 3F). We observed similar results with assays for *L. longipalpis* α-glucosidase (data not shown). We observed in the assays for *R. prolixus* β-glucosidase, β-galactosidase, and α-fucosidase an initial lag phase in the first 5 min but, after that, linearity was restored (Figures 3C,D,F).

**Figure 4:**
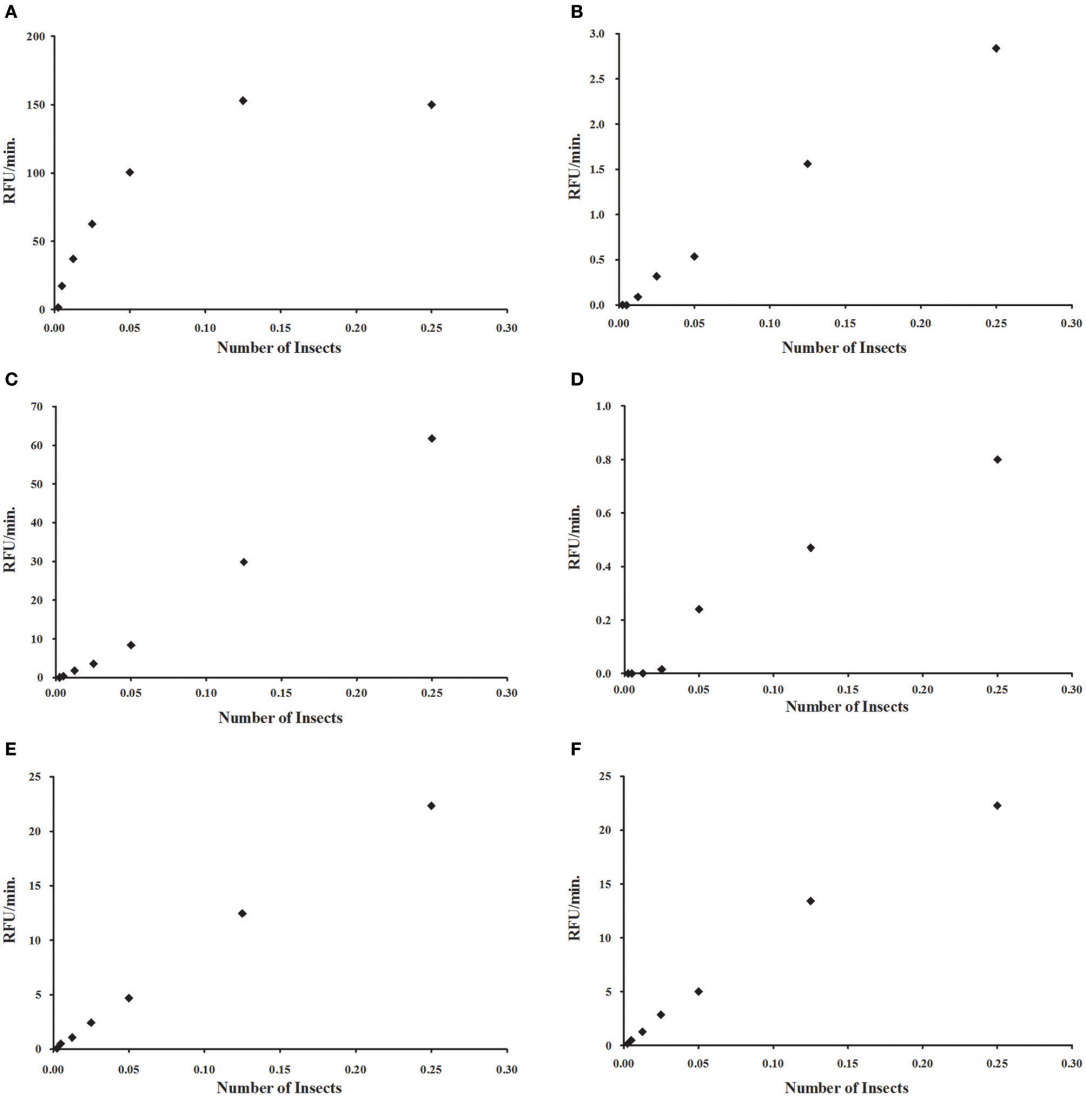
**Relationship between measured activity and protein concentration for different glycosidases, obtained by serial dilution of a sample prepared to include 0.25 insect equivalent per reaction mixture. (A)** α-glucosidase; **(B)** α-mannosidase; **(C)** β-glucosidase; **(D)** β-galactosidase; **(E)** β-N-acetyl-glucosaminidase; **(F)** α-fucosidase. For **(A)**, due to the hyperbolic profile, samples were further diluted to obtain a linear relationship.

Similarly to what was observed with the commercial α-glucosidase, we tested if assays using intestinal homogenates of *R. prolixus* show proportionality between the measured activity and the protein content of the assay (Figure 4). In the case of α-glucosidase, a strong deviation from linearity was observed in higher concentrations of enzyme (Figure 4A). However, we observed linearity for in a wide range of concentrations for α-mannosidase (Figure 4B), β-glucosidase (Figure 4C), β-galactosidase (Figure 4D), β-N-acetyl-glucosaminidase (Figure 4E), and α-fucosidase (Figure 4F). Similar results were obtained for *L. longipalpis* α-glucosidase (data not shown).

**Figure 5:**
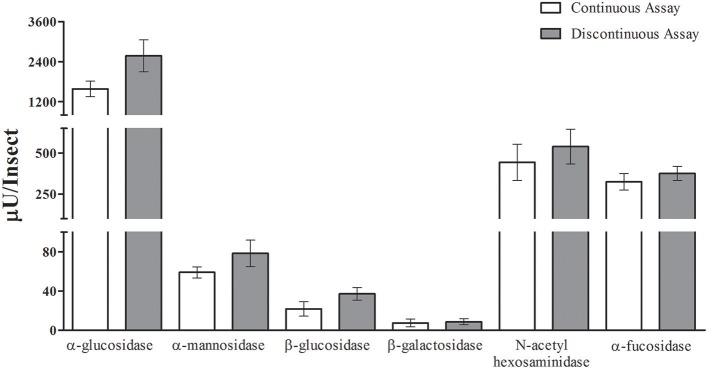
**Comparison of activity measurements obtained with continuous or discontinuous protocols**. Activity of six different glycosidases was measured in five different samples from the *Rhodnius prolixus* anterior midgut tissues of six insects each. No statistically significant difference was observed between the results obtained with both techniques.

**Figure 6:**
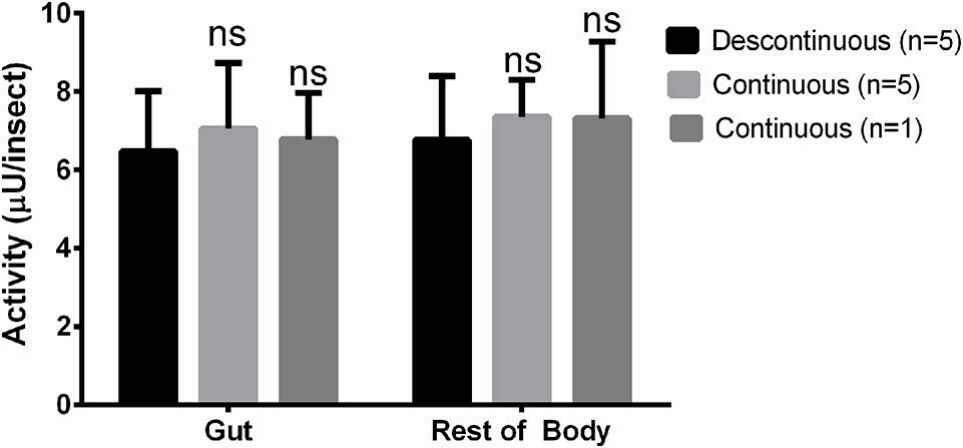
**Comparison of activity measurements obtained with continuous or discontinuous protocols in samples obtained from the sand fly ***Lutzomyia longipalpis*****. Activity of α-glucosidase was measured in different samples from guts or rest of bodies of one or five insects each. No statistically significant difference was observed between the results obtained with both techniques.

After that, we compared the results obtained with the continuous assay to measurements using the traditional discontinuous protocol (interruption by adding carbonate buffer pH 10 in excess). For doing that, we assayed the same samples of anterior midgut tissue homogenates of *Rhodnius prolixus* for the six glycosidases anteriorly standardized using both protocols. The detected activity amounts of α-glucosidase, α-mannosidase, β-glucosidase, β-galactosidase, β-N-acetyl-hexosaminidase and α-fucosidase activities obtained with the continuous assay were statistically similar to the activities detected using the discontinuous protocol (Figure 5). A similar result was obtained when comparing the α-glucosidase activities of *L. longipalpis* tissues measured with both techniques. Surprisingly, the continuous technique allowed us to measure confidently the activity of only one isolated sand fly midgut, which would be impossible to perform with the discontinuous technique (Figure 6). In fact, using the same experimental conditions, discontinuous assays with single fly samples failed to detect (data not shown) the α-glucosidase activity which is observed using samples from pooled insects (Figure 6).

**Figure 7:**
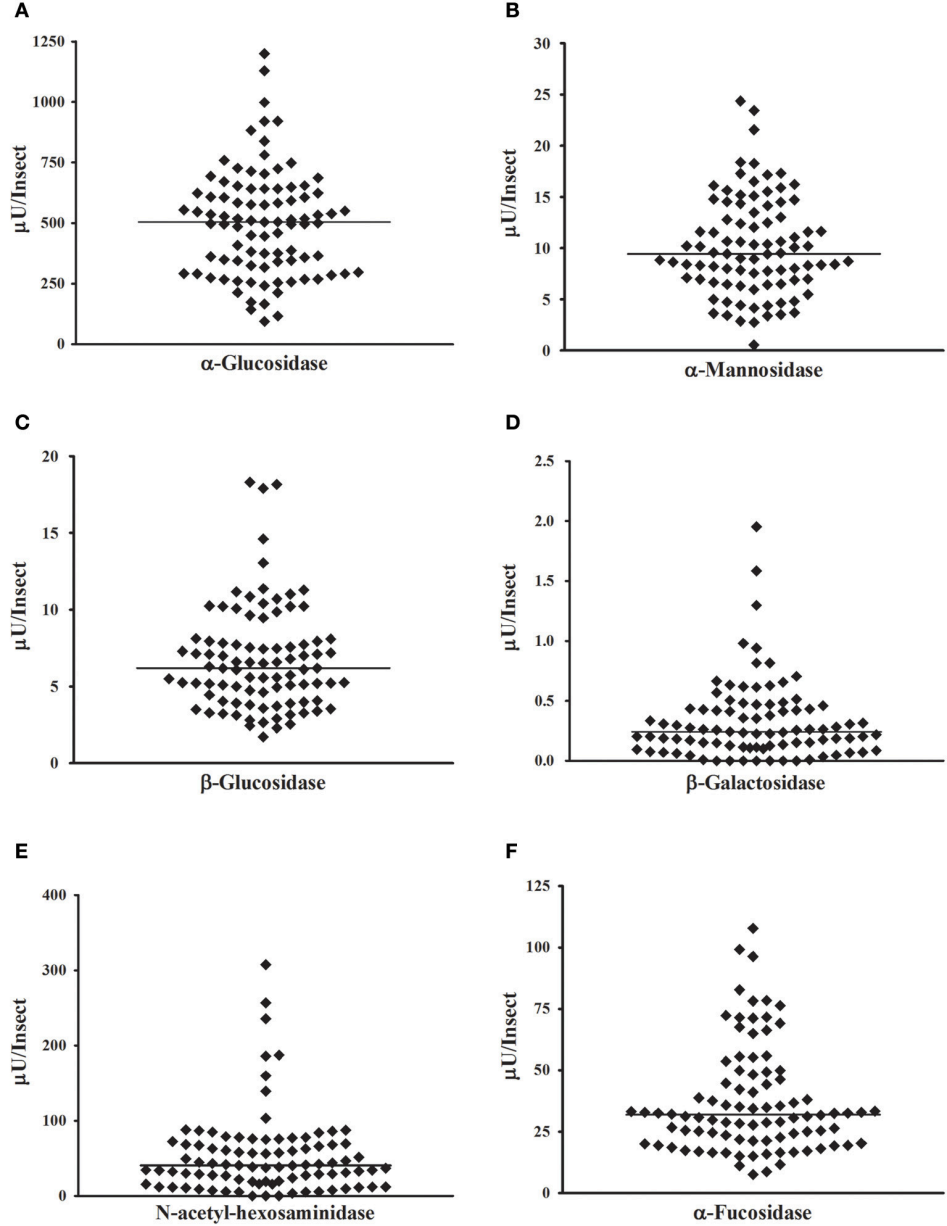
**High-throughput measurement of glycosidase activities in samples obtained from one insect each**. Samples were prepared from 90 *Rhodnius prolixus* anterior midgut tissues, and all determinations were performed in the same day. **(A)** α-glucosidase; **(B)** α-mannosidase; **(C)** β-glucosidase; **(D)** β-galactosidase; **(E)** β-N-acetyl-glucosaminidase; **(F)** α-fucosidase.

After validation of the continuous protocol with our biological samples, we decided to apply the continuous technique to the high-throughput screening of glycosidase activities in individual insect samples. For that, we dissected 90 *R. prolixus* anterior midguts and submitted individual tissue homogenates to the continuous glycosidase assays in the conditions established above. To avoid variations in activity due to different ages or nutritional status, we used synchronized insects that were kept starving for 40 days. The starvation was also performed with the intention of simulating the poor nutritional status that is common in field-collected triatomines. That allowed us to measure the activities of the different six glycosidases in all individual samples in few hours. Results are summarized in Figure 7. We observed a huge variation of activity among individual samples, with more than two orders of magnitude of variation in some cases (Figure 7). However, for all activities tested the distribution of activities in the population allowed us to observe a typical range of activities for each enzyme, as well as a more frequent activity range value, which is typical for each enzyme tested.

**Figure 8:**
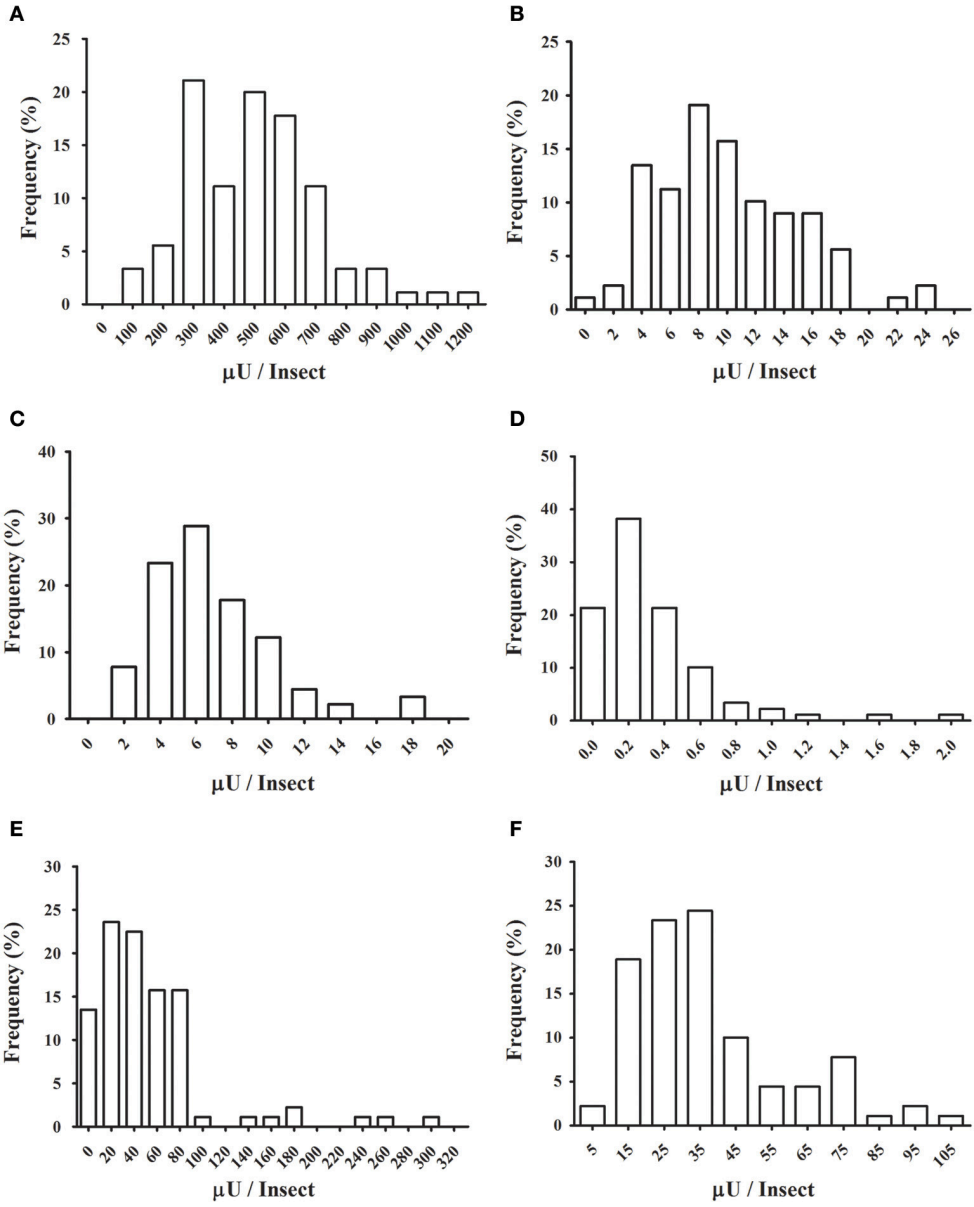
**Frequency distributions of glycosidase activities in the anterior midgut tissue across the population of ***Rhodnius prolixus*** assayed in Figure 7. (A)** α-glucosidase; **(B)** α-mannosidase; **(C)** β-glucosidase; **(D)** β-galactosidase; **(E)** β-N-acetyl-glucosaminidase; **(F)** α-fucosidase.

We decided to analyze the distribution of activities for each enzyme, to test if these activities have a normal distribution across the population. Frequency histograms are presented in Figure 8, and a resume of statistical data analysis is presented in Supplementary Table 1. Normality was observed only for α-glucosidase (Figure 8A) and α-mannosidase (Figure 8B). β-Glucosidase (Figure 8C), β-galactosidase (Figure 8D), β-N-acetyl-glucosaminidase (Figure 8E), and α-fucosidase (Figure 8F) presented non-normal behaviors, with right-skewed and heavy-tailed distributions (Supplementary Table 1).

**Figure 9:**
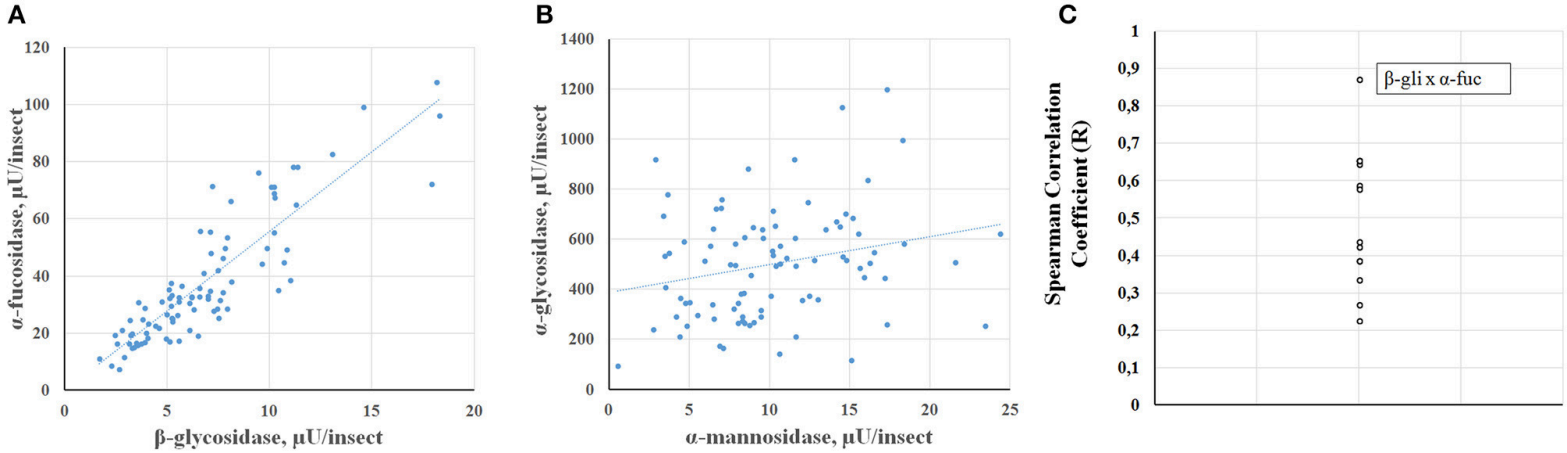
**Correlation of different glycosidase activities obtained in ***Rhodnius prolixus*** samples from Figure 7. (A)** Scatter chart of α-fucosidase vs. β-glucosidase data (*R* = 0.87). **(B)** Scatter chart of α-glucosidase vs. α-mannosidase data (*R* = 0.22). **(C)** Spearman correlation coefficients for all comparisons (see Supplementary Table 2).

Another type of analysis that the high throughput assay allows is to observe the correlation between enzyme activities using a high number of individuals for the comparison. In general, all glycosidase activities showed a significant correlation to the other glycosidases assessed, based on Spearman correlation coefficients (Supplementary Table 2). Nevertheless, some activities showed strong correlations between each other, while other activity comparisons showed poor correlations, as can be observed case by case in scatter plots (Figures 9A,B, respectively) or by comparison of the Pearson correlation coefficients for each comparison (Figure 9C). In this respect, the best correlation observed was between α-fucosidase and β-glucosidase.

## Discussion

The aim of this work was to evaluate the possibility of following glycosidase activities by the continuous release of the methylumbelliferone group in acidic pHs. Methylumbelliferyl-based substrates have been used for a wide range of enzyme activities, but the dependence of the fluorescence on ionization of the group at high pHs has led to obligatory discontinuous assays for the majority of cases (Yang and Hamaguchi, 1980; Hoppe, 1983). Particularly, most insect glycosidases have optimal activities in acidic pHs (Gontijo et al., 1998; Terra and Ferreira, 2005; Jacobson et al., 2007; Cançado et al., 2008; Moraes et al., 2012; Tamaki et al., 2014; Moreira et al., 2015). In this way, the development of substrates with groups that fluoresce in acidic pHs would be greatly beneficial for the study of those enzymes. Despite that, we were able to detect methylumbelliferone even at pHs as acidic as 2 and standardize glycosidase assays at pH 4.5 for *R. prolixus* enzymes. It is important to notice that the application of an enzyme assay will always depend on the specificity of the target enzyme. For example, several insect glycosidases have no activity against synthetic substrates, being able to hydrolyze only their natural substrates, as cellobiose or lactose (Terra and Ferreira, 1994).

In general, linear assays were obtained for all enzymes tested, both in *R. prolixus* and *L. longipalpis* samples. Proportionality of the amount of product released with time is a key factor for the correct measurement of enzyme activities and kinetic parameters, as the main kinetic approaches rely on the measurement of initial velocities of reaction between enzyme and substrate. For discontinuous assays, this implies in the necessity of obtaining several data points and testing for linear correlation of the data. This result in using higher amounts of sample, reagents and time for confirmation of this basic assumption, and result in most cases in a higher occurrence of experimental errors and deviations. The continuous protocol used here to bypass all these difficulties, and in most cases, we were able to confidently measure the initial velocity of reaction in < 10 min.

Another important factor in the measurement of enzyme activities is a proportionality of product release with protein concentration. In this paper, the appropriate assay conditions regarding sample concentration for all enzymes studied were established. For most cases, the observation of a linear correlation between product release and sample concentration was straightforward, with the sole exception of *R. prolixus* α-glucosidase. For this enzyme, inhibition was observed when using high concentrations of the sample. This result might be caused by the presence of inhibitors in the sample, degradation by proteases or by retroinhibition from products released at high concentrations of enzyme.

Physiological inhibitors derived from food molecules are common in insect gut homogenates and, additionally, some glycosidase inhibitors might have important roles in insect-plant and insect-microbial interactions (Terra and Ferreira, 1994). Digestion of bacteria seems to be a necessary step to cope with the paramount bacterial growth which occurs in the anterior midgut after a blood meal. Therefore, it is possible that these glycosidases are being naturally exposed to alternative substrates of bacterial origin. Importantly, α-glucosidase in *R. prolixus* is a particular enzyme of the perimicrovillar membrane with the additional catalytic role of production of hemozoin, an important physiological step in the detoxification of the pro-oxidant heme molecule which is abundant in the blood meal (Silva et al., 2007; Mury et al., 2009). In this way, *R. prolixus* α-glucosidase might have the capacity of binding to different molecules beyond substrates with sugar moieties, as the heme group (Mury et al., 2009), and these molecules might behave as the putative inhibitors that are present in our homogenates.

Another possible source of non-linearity is the proteolytic attack by endogenous proteases. Proteolysis might seem unlikely in the case of samples from *R. prolixus* anterior midgut because this organ has been traditionally considered as a storage compartment and devoided of proteases. However, recent work described important transcription of several proteases in this tissue (Ribeiro et al., 2014). In this case, it was postulated that those enzymes might be secreted in that organ as zymogens, and it is possible that those enzymes are being activated in our assay conditions. In this case, it should be considered that nonlinearity was observed only for α-glucosidase, and it seems unlikely that proteolysis would affect this enzyme preferentially and not the other glycosidases studied here.

Considering retroinhibitions, the two products generated in our α-glucosidase assay are glucose and methylumbelliferone. Inhibition by glucose has been described in a variety of α-glucosidases (Schomburg et al., 2002). No studies regarding the effects of methylumbelliferone on α-glucosidases were performed yet, but some insect glycosidases are inhibited by hydrophobic compounds, mainly aglycones derived from plant glycosides (Hong et al., 2013; Tan et al., 2013). All these possibilities should be considered in future studies of *R. prolixus* α-glucosidase. Despite that, the continuous assay allowed us to find an appropriate sample dilution quickly, by serial dilution and concomitant measurement of a wide range of concentrations of the sample. In this respect, the continuous assay might be valuable for the standardization of assay conditions and characterization of glycosidase activities from unknown samples.

Importantly, the continuous measurement resulted in activity values which are statistically similar to those obtained with the discontinuous protocol from the same samples. Activity values obtained using both techniques were indistinguishable for most of the enzymes studied, with the sole exception of *R. prolixus* α-glucosidase. In this case, values obtained with the continuous assays were somewhat lower than those obtained with the discontinuous assay, but the difference was not statistically significant. This minor artifact does not seem to be related to the specificity of the enzyme because *L. longipalpis* α-glucosidase did not show this behavior. Regardless, values obtained with the continuous assay seem to be reliable, and that encouraged us to use this technique for further studies of these enzyme activities.

Insect glycosidases have been extensively described, studied, and characterized, but the traditional outcome of glycosidase measurements consists in mean activities from samples obtained from pools of several individuals. Sample pooling is firstly a consequence of the limited amount of protein that is recovered from small insects like flies, ants, or beetles. Our approach allowed us for the first time to take a glance in the individual variability that might be hidden in a mean value from a pooled sample. In this case, a wide range of values might correspond to the activities of specific glycosidases, with a considerable presence of outliers, which might distort the mean that is obtained in a pooled sample.

Besides that, the determination of glycosidase activities in samples from individuals allowed us to study the populational distribution of these activities. All activities studied in that regard (six different glycosidases in *R. prolixus* anterior midgut) showed mono-modal frequency distributions, suggesting that they were recorded in a relatively homogenous population. We had the concern of analyzing insects that were synchronized and starved for a long period, to avoid interferences due to different ages or nutritional status, which can severely affect the fitness and physiological characteristics of *R. prolixus* (Díaz-Albiter et al., 2016). However, normal frequency distributions only for two of the activities studied (namely α-glucosidase and α-mannosidase) were observed. The other four activities (β-glucosidase, α-fucosidase, N-acetyl-hexosaminidase, and β-galactosidase) showed non-normal distributions with significant right skewness and kurtosis. Skewed distributions in enzymatic activities might arise from the fact that they are lower-bounded, as there are no negative values. However, a correlation between higher ranges of activity values and normality was not observed, which means that skewness in the non-normal distributions might have other reasons, probably of biological nature. It is important to observe that the non-normal curves presented significant high values for both right skewness and kurtosis. This fact results from the presence of insects with very high activity values. Those exceptional or unusual data points are easily observed in scatter plots like Figure 7 or frequency histograms as Figure 8.

A straightforward consequence of the distributions observed is that for future studies, parametric tests for comparisons between samples and further analysis should be used only for the study of *R. prolixus* α-glucosidase and α-mannosidase. For the other four *R. prolixus* glycosidases non-parametric statistics is recommended. It has been argued that for studies with a high number of samples (above 100) the distinction between parametric or non-parametric is not critical for most analysis (Ghasemi and Zahediasl, 2012). Nevertheless, this is not the case for the majority of insect glycosidase screenings, and the description of non-normal activity distributions might pave the way for more rigorous studies of insect glycosidases. Another consequence of non-normality is that for the distributions with high levels of skewness and heavy tails, the median better reflects the observations than the mean. However, as most observations in the literature use the mean as a reference, this could lead to misinterpretation of results. Non-normal distributions might be an additional reason for the large errors of the mean (~20%) which are common in the descriptions of gut enzymatic activities in insects because they tend to have higher standard deviations than normal ones. It is important to consider that the distributions we have observed came from a limited data set (*N* = 90) and that it might be very interesting to corroborate them in the future with more extensive samplings.

An interesting feature that the individual measurement of glycosidase activities allowed us to assess is the possibility of a correlation between enzymes when looking at different insects across the population. This approach allowed the observation of a positive correlation between all the measured glycosidase activities. We expect a basal level of correlation in all comparisons because all activities would be dependent on the protein content of samples. Nevertheless, unusually high levels of correlation between some glycosidases were registered, which might reflect physiological functional groups of enzymes working in concert against the same substrate. Of special interest is the grouping between β-glucosidase and α-fucosidase. We need further studies to understand the relevance of these correlations, both at the molecular and physiological levels.

To our knowledge, this work is the first high-throughput screening of insect glycosidases performed at the individual level. It is important to notice that these enzymes are key factors in pathogen-vector and insect-plant interactions. Some glycosidases have been described as markers for insect resistance against plant defenses (Terra and Ferreira, 2005). We believe that the standardization of such high-throughput glycosidase assays will benefit not only the theoretical studies about the physiological role of these enzymes but also the monitoring of populations of sylvatic insects, agricultural pests, and disease vectors with a more powerful biochemical toolbox.

## Author contributions

Conception and design of the work: GP, CM, FG. Obtainment of experimental data: GP, JP, SC. Data analysis: GP, JP, SC, FG. Writing and revision of the manuscript: GP, JP, SC, PA, EG, CM, FG.

### Conflict of interest statement

The authors declare that the research was conducted in the absence of any commercial or financial relationships that could be construed as a potential conflict of interest. The reviewer MP declared a past co-authorship with several of the authors SC, PA, and EG to the handling Editor, who ensured that the process met the standards of a fair and objective review.
