# Supplementary material for: Standardization of a Continuous Assay for Glycosidases and Its Use for Screening Insect Gut Samples at Individual and Populational Levels
Source: Front Physiol. 2017 May 12;8:308. doi: 10.3389/fphys.2017.00308 (PMC5427678; doi:10.3389/fphys.2017.00308)
Supplement: Supplementary file 1 [file Table1.PDF]

Supplementary Material

Standardization of a continuous assay for glycosidases and its use for screening insect gut samples at individual and populational levels.

Gerson S. Profeta, Jessica A. S. Pereira, Samara G. Costa, Patricia Azambuja, Eloi S. Garcia, Caroline S. Moraes*, Fernando A. Genta[1]

*** Correspondence:** Caroline S. Moraes: carolinemoraes83@gmail.com

Supplementary Table 1. Statistical parameters of activity distributions for *Rhodnius prolixus* glycosidases

Glycosidase	Relative Skewness	Relative Kurtosis
α-fucosidase	1.21	1.00
α-glucosidase	0.63	0.60
β-glucosidase	1.33	2.26
β-galactosidase	2.34	7.62
α-mannosidase	0.60	0.21
N-acetyl- β-hexosaminidase	2.45	7.33

frontiers

frontiers
