# Supplementary material for: Standardization of a Continuous Assay for Glycosidases and Its Use for Screening Insect Gut Samples at Individual and Populational Levels
Source: Front Physiol. 2017 May 12;8:308. doi: 10.3389/fphys.2017.00308 (PMC5427678; doi:10.3389/fphys.2017.00308)
Supplement: Supplementary file 2 [file Table2.PDF]

Supplementary Material

Standardization of a continuous assay for glycosidases and its use for screening insect gut samples at individual and populational levels.

Gerson S. Profeta, Jessica A. S. Pereira, Samara G. Costa, Patricia Azambuja, Eloi S. Garcia, Caroline S. Moraes*, Fernando A. Genta[1]

*** Correspondence:** Caroline S. Moraes: carolinemoraes83@gmail.com

Supplementary Table 2. Coefficients and *p*-values for Spearman correlation tests between *Rhodnius prolixus* glycosidase activities (white and grey diagonals, respectively). NAH – N-acetyl-β-hexosaminidase.

Glycosidase	α-fucosidase	α-glucosidase	β-glucosidase	β-galactosidase	α-mannosidase	NAH[1]
α-fucosidase		< 0.0001	< 0.0001	< 0.0001	< 0.0001	< 0.0001
α-glucosidase	0.6538	-	< 0.0001	< 0.0001	0.0338	0.0002
β-glucosidase	0.8724	0.6546	-	< 0.0001	0.0002	< 0.0001
β-galactosidase	0.6441	0.4361	0.6547	-	0.0108	< 0.0001
α-mannosidase	0.4231	0.2253	0.3866	0.2689	-	0.0014
NAH	0.5843	0.3855	0.5879	0.578	0.3345	-

frontiers

frontiers
